# Immunological Findings in a Group of Individuals Who Were Poor or Non-Responders to Standard Two-Dose SARS-CoV-2 Vaccines

**DOI:** 10.3390/vaccines11020461

**Published:** 2023-02-16

**Authors:** Qiang Zeng, Xue Yang, Biao-Yang Lin, Yong-Zhe Li, Gang Huang, Yang Xu

**Affiliations:** 1 Health Management Institute, The Second Medical Center & National Clinical Research Center for Geriatric Diseases, Chinese PLA General Hospital, Beijing 100039, China; 2 Outpatient Department, The Second Medical Center & National Clinical Research Center for Geriatric Diseases, Chinese PLA General Hospital, Beijing 100039, China; 3 Institute of Nanotechnology, Zhejiang University, Hangzhou 310058, China; 4 Department of Laboratory Medicine, Peking Union Medical College Hospital, Beijing 100730, China; 5 Laboratory of Special Diagnosis & Shanghai Key Laboratory of Molecular Imaging, Shanghai University of Medicine and Health Sciences, Shanghai 201318, China

**Keywords:** COVID-19, vaccine, non-responders, international standard, B cell immunity, BAU/mL, cut-off, IgG

## Abstract

Coronavirus disease (COVID-19), caused by severe acute respiratory syndrome coronavirus 2 (SARS-CoV-2), has been declared a pandemic. However, data on the poor or non-responders to SARS-CoV-2 vaccines in the general population are limited. The objective of this study was to comprehensively compare the immunological characteristics of poor or non-responders to SARS-CoV-2 vaccines in the 18–59-year group with those in the ≥60-year group using internationally recognized cut-off values. The main outcome was effective seroconversion characterized by an anti-SARS-CoV-2 spike IgG level of at least a four-fold increase from baseline. Profiling of naïve immune cells was analyzed prior to vaccination to demonstrate baseline immunity. The outcomes of effective seroconversion in patients aged 18–59 years with those in patients aged ≥60 years were compared. The quantitative level of anti-spike IgG was significantly lower in individuals aged ≥60 and men aged 18–59 years. There were 7.5% of poor or non-responders among the 18–59 years and 11.7% of poor or non-responders in the ≥60 years using a four-fold increase parameter. There were 37.0–58.1% with low lymphocyte count (<1000/mm^3^), 33.3–45.2% with low CD4 cell counts (<500/mm^3^), and 74.1–96.8% with low B cell counts (<100/mm^3^) in the non-seroconversion group. An individual with an anti-SARS-CoV-2 spike IgG titer below 50 BAU/mL might be considered a poor or non-responder between 14 and 90 days after the last vaccine dose. Booster vaccination or additional protective measures should be recommended to poor or non-responders as soon as possible to reduce disease severity and mortality.

## 1. Introduction

Coronavirus disease (COVID-19), caused by severe acute respiratory syndrome coronavirus 2 (SARS-CoV-2), has been declared a pandemic. The virus has infected more than 640 million people and caused more than 6.6 million deaths [1]. Since December 2020, the World Health Organization (WHO) recommends vaccination against COVID-19, nine types of COVID-19 vaccines have been included in the emergency use list [2].

Vaccination against COVID-19 is especially important in reducing severe illnesses and mortality. According to the data from the Centers for Disease Control and Prevention (CDC) in 2016–2017, the mortality rate caused by influenza virus was 0.13% [3].

To bring the COVID-19 pandemic under control as soon as possible and ensure that the mortality rate is close to that caused by the influenza virus, the prevention and treatment of children, as well as older adults and immunocompromised people, has emerged as a top priority [4,5,6,7,8]. Sun et al. have reported that hospitalization and severe outcomes were similar in unvaccinated healthy individuals and immunocompromised patients who received two doses of SARS-CoV-2 vaccination in the United States, suggesting that COVID-19 breakthrough infection after vaccination is associated with immune dysfunction. Hospitalization and severe outcomes were 21.1% and 1.9%, respectively, in unvaccinated healthy individuals, and 20.7% and 2.1% in patients with immune dysfunction after 14 days following full vaccination, indicating that an immune barrier is not well established. Therefore, post-vaccination serologic testing (PVST) is necessary to identify immunocompromised individuals without specific immunity so that they can be given additional prophylaxis after full vaccination [8]. This study suggests that PVST helps reduce mortality, demonstrating the importance and urgency of PVST using an international standard.

To date, more than 5 billion people have been vaccinated against COVID-19 [9]. In clinical trials associated with COVID-19 vaccination, the efficacy of COVID-19 vaccination to elicit specific B cell or antibody responses has been reported. Effective immunogenicity or humoral immune response is defined as a ≥4-fold increase in antibody titers from baseline and is considered the gold standard for assessing specific B cells or antibody protection in vaccinated recipients in clinical studies [10,11,12]. In contrast, a poor or non-responder is an individual who demonstrates no effective immunogenicity or humoral immune response despite the completion of the suggested vaccination procedure [13,14].

Non-responders to the hepatitis B vaccine have been described. Szmuness et al. have reported that 7.4% of immunized individuals fail to elicit detectable specific antibodies after two doses of the hepatitis B vaccine, suggesting that there are non-responders in the population [15]. Roome et al. have found that 11.9% of individuals with the hepatitis B vaccine had no or inadequate levels of antibody, suggesting that PVST should be performed at intervals of 30 to 90 days after the last vaccine dose [16].

Repeated poor or non-responders to a third or fourth dose of the SARS-CoV-2 vaccine were first observed in transplant recipients. Caillard et al. reported a cohort of 92 renal transplant recipients who did not have effective seroconversion after the third dose of mRNA vaccines [17]. Furthermore, there were 52.9% (18/34) of poor or non-responders to BNT162b2 vaccine and 48.3% (28/58) of poor or non-responders to the mRNA-1273 vaccine after the fourth dose of mRNA vaccines, showing that PVST should be performed after the third or fourth vaccine dose.

Poor or non-responders to the SARS-CoV-2 vaccine are vulnerable populations with poor outcomes and high mortality rates. Chukwu et al. reported clinical findings in a group of kidney transplant recipients who received two doses of the SARS-CoV-2 vaccine [18]. There were 22 breakthrough infections and three deaths after vaccination, including 77% (17/22) infections and 13.6% (3/22) deaths in the poor or non-responder group and only 23% (5/22) infections and 0% (0/22) deaths in the responder group [18]. Therefore, there is an urgent need to identify SARS-CoV-2 vaccine poor or non-responders in the vulnerable population to reduce severe COVID-19 and mortality.

During the promotion of vaccination, several factors affecting the response to the SARS-CoV-2 vaccine were considered, especially the reduced response to the SARS-CoV-2 vaccine in children, older adults, and immunocompromised populations. It has been documented that 5–10% of poor or non-responders to hepatitis B vaccines are healthy individuals [19]. However, the immunological characteristics of poor or non-responders to SARS-CoV-2 vaccines in clinical trials remain limited [20,21,22,23,24].

In December 2020, the WHO issued an International Standard (IS) for the quantification of anti-SARS-CoV-2 immunoglobulin for PVST [25,26]. This standard provides a unified benchmark for effective antibody protective concentrations after vaccination. 

Zhang et al. reported a cohort of 75 healthy individuals aged 18–59 years who received two doses of the inactivated SARS-CoV-2 vaccine [27]. There were 9.3% (7/75) of poor or non-responders to the SARS-CoV-2 vaccine in the cohort study, and a low lymphocyte count was a risk factor. However, internationally recognized cut-offs according to the WHO IS and data from older adults (≥60 years) have not been reported. 

This clinical study aimed to describe the immunological characteristics of 627 individuals aged 18–86 years who volunteered to participate in the COVID-19 vaccination and comprehensively compare the poor or non-responders in the age 18–59 years with those aged ≥60 years to explore internationally recognized cut-offs. Profiling of naïve immune cells was performed prior to vaccination to demonstrate the baseline immunity. After two doses of vaccination, the antibody titer increased by ≥ 4 times from baseline as the gold standard. Furthermore, data using the WHO IS cut-off were analyzed to provide insights for identifying poor or non-responders and improving the efficacy of vaccines, which helps to reduce breakthrough infections after vaccines, and ultimately reduces disease severity and mortality.

## 2. Methods

### 2.1. Study Design and Participants

We conducted a prospective, observational study of individuals aged 18–86 years who were part of the antibody immunity evaluation after SARS-CoV-2 vaccination in Beijing, China. The study cohort comprised 627 individuals who underwent physical examinations from 1 April to 30 September 2021, in the regular and geriatric clinic of the Health Management Institute, Beijing, China (Figure S1). The protocol and informed consent of the clinical study were reviewed and approved by the institutional ethics committee (No. S2021-481-01) and all study participants provided their written informed consent before screening for eligibility. Inclusion criteria: (1) adult individuals who received physical examinations from 1 April to 30 September 2021, and (2) individuals who were willing to donate blood samples. Exclusion criteria: (1) individuals who received ≥ 1 dose of SARS-CoV-2 vaccine, (2) positive reverse transcription PCR results for SARS-CoV-2 on nasopharyngeal swabs, and (3) presence of anti-SARS-CoV-2 immunoglobulin (Ig)M/IgG antibodies. Finally, 627 participants were enrolled and completed the study, of which 361 cases (18–59 years, mean 45 years, male 50.7%) completed two doses of HB02 vaccine from the regular clinic, and 266 (60–86 years, mean 67 years, male 51.1%) from the geriatric clinic. Assay operators were all blinded to the participant study. Participants received two intramuscular injections on day 0 and 21, in the deltoid muscle. Each injection contained 4 μg/0.5 mL of Sinopharm COVID-19 vaccine (HB02 strain), which is an inactivated SARS-CoV-2 vaccine from the Sinopharm, China National Pharmaceutical Group Co., Ltd, Beijing, China. Sinopharm has developed two types of inactivated SARS-CoV-2 vaccines, WIV04 and HB02. According to the clinical trial, the inactivated SARS-CoV-2 HB02 strain is more potent than the WIV04 strain [24]. Blood was collected from participants on day 0 and 21 and at the interval of 14 to 90 days after the second dose. The observed humoral response was likely generated by the HB02 vaccine, not through natural infections, because the serologic screening and nucleic acid testing were performed to try to ensure that none of the participants was infected with SARS-CoV-2 before enrollment. In addition, no new COVID-19 cases were reported at the study area and no participant developed any symptoms of SARS-CoV-2 infection during the study period. A vaccine breakthrough infection from the Centers for Disease Control and Prevention is defined as a positive test result for the SARS-CoV-2 in the respiratory specimen of a recipient more than 14 days after completing the recommended dose of COVID-19 vaccine. Based on the above definitions, we conducted post-vaccination serologic testing on day 14 after the second dose of vaccine. The pre-second-dose titers were determined on day 21 before the second dose and the post-second-dose titers were determined at intervals of 14 to 90 days after the second dose.

### 2.2. Enzyme-Linked Immunosorbent Assay (ELISA) of Immunoglobulin (Ig)

Levels of specific antibodies were determined using two different ELISAs: an in-house assay that used SARS-CoV-2 RBD/Spike protein (Cat. #Z03479, Genscript Biotech, Piscataway, NJ, USA) as an antigen and CR3022 antibody (Cat. #ZHU1077, Sigma-Aldrich, St. Louis, MO, USA) as a positive control (CR3022 antibody showed RBD cross-reactivity only in SARS-CoV-1 and SARS-CoV-2, and was minimally affected by SARS-CoV-2 variants), and a commercial assay (COVID-SeroKlir, Kantaro SARS-CoV-2 IgG Antibody Kit, Cat. #COV219-100, Kantaro Biosciences, New York, NY, USA). For in-house assay, microtiter plates were coated with 50 ng/well of target protein overnight at 4 °C. Plates were then blocked for 2 h at 37 °C using 200 μL of 5% non-fat milk in phosphate-buffered saline (PBS). Serum samples were then diluted 1:50 using 1% non-fat milk in PBS and 100 μL of each sample was applied to the coated ELISA plate and incubated for 2 h at 37 °C before three more washes with PBST (PBS with 0.1% Tween 20, Sigma-Aldrich, St. Louis, MO, USA). Samples were then incubated for 1 h at room temperature with a 1:2000 dilution of anti-human IgG horseradish peroxidase (HRP) or 1:2000 dilution of anti-human IgM HRP in the dark (Sigma Aldrich, St. Louis, MO, USA). Plates were washed three times, and 100 μL of the TMB/E substrate solution (Millipore, Burlington, MA, USA) was added to each well. Finally, the reaction was stopped with 1 M H_2_SO_4_, and the optical density (OD) at 450 nm was measured. A negative serum control was run each time with the assay. A sample is positive if its adjusted OD value (OD of test–OD of control) exceeds the mean plus 3 standard deviations of the normal controls. For each sample, the ELISA end point titer was calculated using a four-parameter logistic curve fit to calculate the reciprocal serum. For commercial assay, anti-SARS-CoV-2 spike IgG testing was performed using a kit with Food and Drug Administration (FDA) Emergency Use Authorization according to the manufacturer’s instructions. The antibodies to SARS-CoV-2 spike protein were detected using an established two-step ELISA (COVID-SeroKlir Kantaro SARS-CoV-2 IgG Ab Kit, New York, NY, USA). The assay showed a performance of a specificity of 99.8% and a sensitivity of 97.8%. The concentration of the WHO international standard (IS) for anti-SARS-CoV-2 immunoglobulin (#20/136, GISA Biosciences, Fairfield, CA, USA) is 1000 BAU/mL (for binding antibody assays) or 1000 IU/mL (for neutralizing antibody assays). The WHO IS concentrations in the experiment were 1000 BAU/mL, 500 BAU/mL, 250 BAU/mL, 125 BAU/mL, 62.5 BAU/mL, 31.3 BAU/mL, 15.6 BAU/mL, and 7.8 BAU/mL. The data from our in-house assay and a quantitative commercial kit (COVID-SeroKlir Kantaro SARS-CoV-2 IgG Ab Kit, New York, NY, USA) were similar (data not shown); however, only data from the quantitative commercial kit were presented because this kit has been extensively evaluated in many clinical studies.

### 2.3. Flow Cytometry Analysis

All blood samples were analyzed using a FACSVerse™ flow cytometer (BD Biosciences, Franklin Lakes, NJ, USA), and the flow cytometry gating strategy has been described in our previous publication [6]. Briefly, all the antibodies (CD3-FITC, CD45-PerCP-Cy™5.5, CD19-APC, CD16/56-PE, CD4-PE-Cy™7, and CD8-APC-Cy7) were supplied by BD Biosciences (Franklin Lakes, NJ, USA). Blood samples were collected and stained according to the manufacturer’s instructions. Red cell lysis buffer was added to each sample and incubated for 10 min, then washed using a Sorvall cell washer (Thermo Fisher Scientific, Waltham, MA, USA). Cells were blocked with FcR blocking reagent (Miltenyi Biotec, Gaithersburg, MD, USA) for 30 min at 4 °C to reduce the nonspecific binding of antibodies to human FcR and then washed with phosphate-buffered saline (PBS). The cells were then stained with antibodies using a fixable dead cell stain kit (Invitrogen, Waltham, MA, USA) for 30 min at 4 °C, resuspended in PBS, and analyzed by flow cytometry (FACSVerse™ flow cytometry, BD Biosciences, Franklin Lakes, NJ, USA). Calibration and quality control of the instrument were performed daily using eight-color setup beads (BD Biosciences, Franklin Lakes, NJ, USA). All specimens were analyzed in duplicate with a coefficient of variation (CV) < 5% by two independent technicians under the inter-laboratory quality control. The experiments were repeated if the results exhibited a CV > 5%, according to the instructions of BD Biosciences. Data were analyzed using FlowJo software (version 10, Tree Star, Ashland, OR, USA).

### 2.4. Statistical Analysis

All statistical analyses were conducted using GraphPad Prism version 9.3.1 software (GraphPad Software™, San Diego, CA, USA) and Statistical Package for the Social Sciences 23.0 (SPSS 23.0, IBM Corporation, Armonk, NY, USA). Effective seroconversion was defined as an antibody titer ≥4 times from baseline. Categorical variables are expressed as frequency rates and percentages, and continuous variables are expressed as mean, median, and interquartile range (IQR) values. Means for continuous variables were compared using independent group t-tests when the data were normally distributed; otherwise, the Mann–Whitney test was used. Data (non-normal distribution) from repeated measures were compared using a generalized linear mixed model. Proportions for categorical variables were compared using the chi-square test, although Fisher’s exact test was used when the data were limited. All tests were two-sided, and a *p*-value less than 0.05 was considered statistically significant. All statistical results were interpreted as exploratory or descriptive.

## 3. Results

### 3.1. Baseline Immunological Characteristics of Individuals Prior to Vaccination

**Figure 1:**
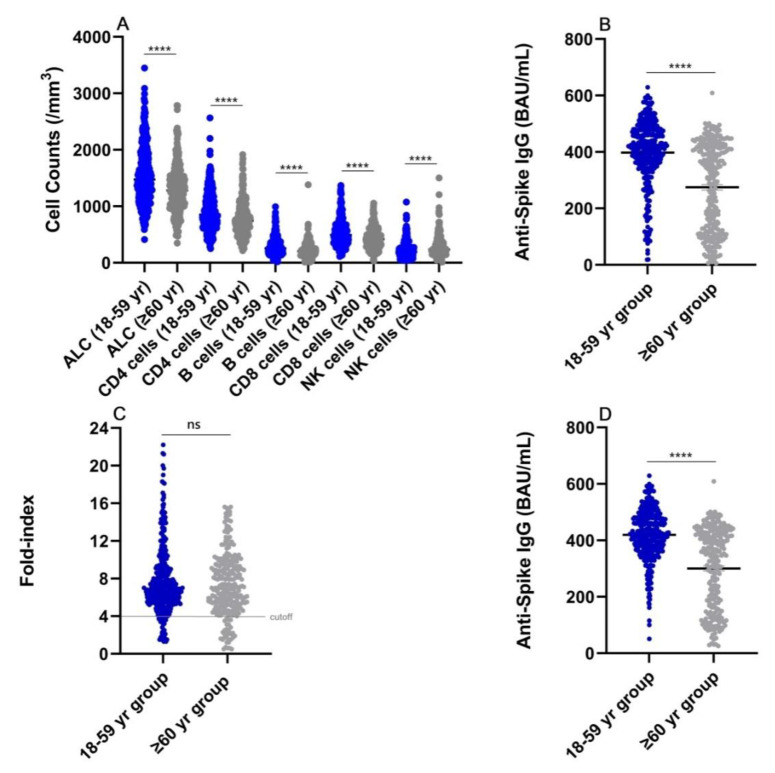
Immunological characteristics of 627 individuals. (**A**) Naïve cellular immunological parameters of the 627 cases who received physical examinations. These naïve immune cells wane significantly, while the natural killer (NK) cell counts increase significantly in older adults. ALC, absolute lymphocyte count. (**B**) The anti-spike IgG levels after complete vaccination of the 627 cases. The quantitative level of the anti-spike IgG is significantly lower in the ≥60-year group (median 307.2, IQR 118.2–417.3 BAU/mL) than that in the 18–59-year group (median 416.8, IQR 355.7–479.2 BAU/mL, *p* < 0.0001). Mean and standard error of the mean (SEM) are shown. (**C**) The vaccine-induced responses using at least a four-fold increase in antibody titer from baseline in 627 cases. There are 7.5% of poor or non-responders (fold-index < 4) among the 18–59-year group and 11.7% in the ≥ 60-year group. The level of anti-spike IgG ranges (the 1st–99th percentile) for responders (fold–index ≥4) are 43.9–592.0 BAU/mL in combination of the 18–59-year and the ≥60-year groups. A cut-off line at fold-index 4 is shown. (**D**) In the responder group (fold-index ≥ 4), levels of anti-spike IgG for the 1st–99th percentile are 131.8–592.3 BAU/mL in the 18–59-year group and 29.7–500.9 BAU/mL in the ≥ 60-year group. Mean and SEM are shown. **** *p* < 0.0001. ns, not significant.

There were 627 individuals with anti-RBD/Spike IgM and IgG negative prior to vaccination, 42.4% (266/627) aged ≥60 years, and 50.9% (319/627) males enrolled in the study (Table 1). In the 18–59-year group, the median (interquartile range [IQRs]) absolute lymphocyte count (ALC), CD4 cell count, CD8 cell count, B cell count, and natural killer (NK) cell count were 1476 (1168–1875), 851 (677–1151), 490 (357–632), 256 (179–367), and 193 (141–287)/mm^3^, respectively. On the contrary, in the ≥60-year group, the respective medians (IQRs) were 1281 (1023–1520), 747 (562–955), 418 (288–544), 204 (138–303), and 234 (162–355)/mm^3^ (Table 1). The number of naïve lymphocytes, CD4 cells, CD8 cells, and B cells was significantly reduced in the older adult (≥60 years) population than that in the 18–59-year population (*p* < 0.0001). Hence, these naïve immune cells wane significantly, whereas NK cell counts increase significantly in older adults (Table 1, Figure 1A).

### 3.2. Characteristics of Humoral Response after Complete Vaccination

We analyzed anti-spike IgG levels after completing two doses of vaccination in 627 cases (Table 1). Post-vaccination testing was performed 14–90 days after the second vaccine dose. The quantitative level of the anti-spike IgG was significantly lower in the ≥60-year group (median 307.2, IQR 118.2–417.3 BAU/mL) than that in the 18–59-year group (median 416.8, IQR 355.7–479.2 BAU/mL) (Table 1, Figure 1B). The reference ranges (the 2.5th–97.5th percentile) were 88.9–576.2 BAU/mL in the 18–59-year group and 27.7–491.0 BAU/mL in the ≥ 60-year group at intervals of 14–90 days after complete vaccination (Table 2).

### 3.3. Characteristics of Humoral Response in Male and Female after Complete Vaccination

**Figure 2:**
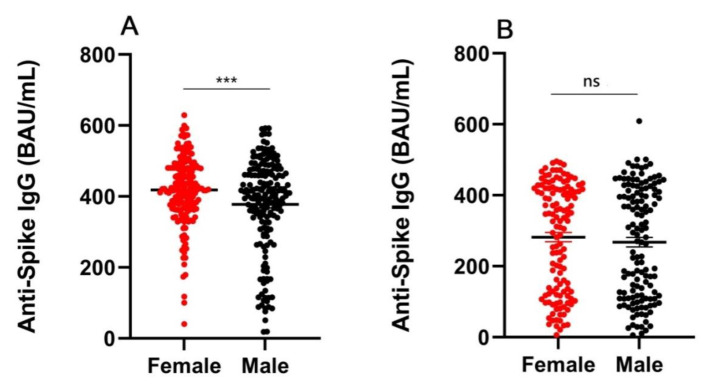
Sex affected anti-spike IgG levels after complete vaccination. (**A**) The quantitative level of the anti-spike IgG is significantly lower in the male group (median 404.9, IQR 326.7–471.7 BAU/mL) than that in the female group (median 421.7, IQR 367.1–480.7 BAU/mL) in the 18–59-year group (*p* = 0.0008). The means and SEM are shown. (**B**) No significant difference in the ≥ 60-year group for the quantitative levels of anti-spike IgG between the male (median 285.4, IQR 113.1–416.3 BAU/mL) and female groups (median 327.5, IQR 126.7–418.2 BAU/mL) (*p* = 0.4517). The means and SEM are shown. IQR, interquartile range; SEM, standard error of the mean. *** *p* < 0.001. ns, not significant.

The quantitative level of the anti-spike IgG was significantly lower in the male group (median 404.9, IQR 326.7–471.7 BAU/mL) than that in the female group (median 421.7, IQR 367.1–480.7 BAU/mL) in the 18–59-year group (*p* = 0.0008) (Figure 2A). There was no significant difference in the ≥60-year group for the quantitative levels of anti-spike IgG between the male (median 285.4, IQR 113.1–416.3 BAU/mL) and female groups (median 327.5, IQR 126.7–418.2 BAU/mL) (*p* = 0.4517) (Figure 2B).

### 3.4. Dynamics of Anti-Spike IgG Levels after Complete Vaccination

**Figure 3:**
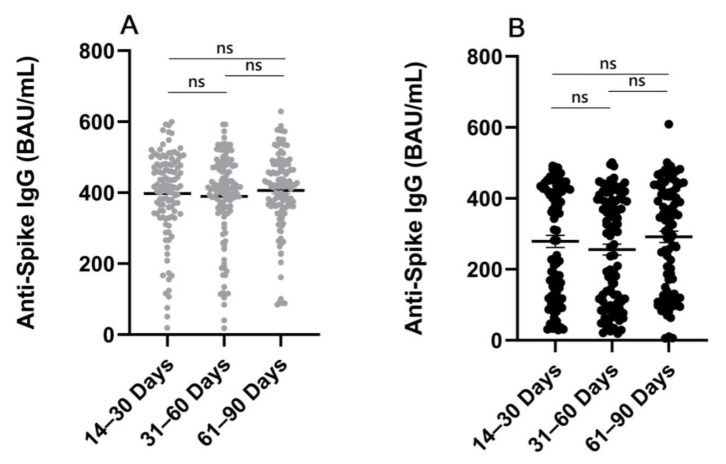
Dynamics of anti-spike IgG levels after complete vaccination. (**A**) There are no significant differences in the quantitative level of anti-spike IgG at different time points (14–30 days, 31–60 days, and 61–90 days) among the 18–59-year group (*p* > 0.1 between groups). The means and SEM are shown. (**B**) There are no significant differences in the quantitative levels of anti-spike IgG at different time points (14–30 days, 31–60 days, and 61–90 days) in the ≥ 60-year group (*p* > 0.1 between groups). The means and SEM are shown. SEM, standard error of the mean. ns, not significant.

The overall distribution of mean anti-spike IgG levels in serum samples at different time points (14–30, 31–60, and 61–90 days) after completion of the second dose of vaccination was uniform. There were no significant differences in the quantitative level of anti-spike IgG at different time points between the 18–59-year (*p* > 0.1 between groups) and ≥60-year groups (*p* > 0.1 between groups) (Figure 3).

### 3.5. Characteristics of Responders and Poor or Non-Responders to the SARS-CoV-2 Vaccine Using Internationally Recognized Standards

Thereafter, we evaluated the vaccine-induced responses based on the post- and pre-second-dose titers, using a four-fold increase parameter (fold-index <4 or ≥4) and the WHO IS (Table 2). Remarkably, there were 7.5% of poor or non-responders (fold-index <4) in the 18–59-year group and 11.7% of poor or non-responders in the ≥60-year group (Table 2, Figure 1C).

In the 18–59-year group, the median (IQR) levels of anti-spike IgG and the reference ranges were 115.8 (88.6–167.8) and 11.3–266.3 BAU/mL with fold-index <4 and 420.8 (369.9–480.6) and 200.7–576.5 BAU/mL with fold-index ≥4, respectively (*p* < 0.0001) (Table 2). In contrast, in the ≥ 60-year group, the median (IQR) levels of anti-spike IgG and the reference ranges were 63.9 (35.1–106.9) and 5.4–317.8 BAU/mL with fold-index <4 and 346.0 (160.4–424.7) and 46.6–491.1 BAU/mL with fold-index ≥4, respectively (*p* < 0.0001) (Table 2). The level of anti-spike IgG ranges (the 1st–99th percentile) for all responders (fold-index ≥4) were 43.9–592.0 BAU/mL in the combination of the 18–59-year and ≥ 60-year groups at intervals of 14–90 days after complete vaccination (Figure 1C).

### 3.6. Relationship between Seroconversion Rate and Baseline Immunity

We observed that the effective seroconversion rate (fold-index ≥4) was significantly related to the levels of certain naïve immune cells before vaccination (Table 2). Particularly, lymphocyte, CD4, and B cell counts were significantly different (*p* < 0.0001) between the fold-index <4 and ≥4 groups. Regarding the CD8 cell count, a significant difference was noted only between the individuals with fold indexes <4 and ≥4 in the 18–59-year group [414/mm^3^ (95% confidence interval [CI] 349–479/mm^3^) vs. 532/mm^3^ (95% CI 508–557/mm^3^), *p* = 0.0081]. However, the CD8 cell count in the ≥60-year group and the NK cell count in both age groups did not show any significant differences.

We further analyzed the non-seroconversion group (fold-index <4) after the second dose (Table 3). There were 37.0–58.1% with low lymphocyte count (<1000/mm^3^), 33.3–45.2% with low CD4 cell counts (<500/mm^3^), and 74.1–96.8% with low B cell counts (< 100/mm^3^), suggesting that low lymphocyte count, low CD4 cell counts, and low B cell counts were risk factors for poor or non-responders to vaccines.

## 4. Discussion

To the best of our knowledge, this is one of the first clinical studies to report poor or non-responders after the administration of two doses of inactivated SARS-CoV-2 vaccines using the WHO IS cut-off value for anti-SARS-CoV-2 immunoglobulin with documented baseline immunity and effective seroconversion using antibody titers ≥4 times from baseline as the gold standard. There were no significant differences in the quantitative level of anti-spike IgG at different time points (14–30, 31–60, and 61–90 days) after administering two doses of the vaccine. Previous clinical trials have demonstrated that individuals produce 100% neutralizing antibodies after an effective seroconversion (a four-fold response post-immunization) and have similar neutralizing capacity against the D614G and B.1.1.7 variants compared with the wild-type virus after receiving two doses of the SARS-CoV-2 HB02 vaccine through cross-reactivity; however, there is low or no neutralizing antibody activity if antibody titers are less than four times from baseline [10,24].

Whether there is a specific B cell or humoral immune response following COVID-19 vaccination is a marker of population immunity [28,29,30]. Typically, healthy individuals with normal immune systems have normal immune cell counts and an effective specific B cell or humoral immune response, defined as a ≥4-fold increase in antibody titers from baseline within 14–90 days of the vaccination schedule. The use of anti-SARS-CoV-2 assays with the use of the WHO IS can facilitate the comparison of the strength of the specific B cell or humoral immune response between individuals, making the data more accurate and providing reliable data for the COVID-19 vaccine booster. Therefore, adequate clinical trials are necessary to assess the immune characteristics of individuals prior to vaccine booster shots, such that mortality in the pandemic may be quickly reduced.

We used an anti-SARS-CoV-2 spike quantitative IgG kit (COVID-SeroKlir Kantaro SARS-CoV-2 IgG Ab Kit) approved by the Food and Drug Administration (FDA) under Emergency Use Authorization (EUA) with the WHO IS. This kit has been extensively evaluated in many clinical studies, including neutralizing antibodies after SARS-CoV-2 infection, immunological memory to SARS-CoV-2, convalescent plasma treatment of severe COVID-19, and antibody responses to mRNA vaccines in healthy people and patients [30,31,32,33,34,35]. After complete two-dose vaccination, the level of anti-spike IgG ranges (the 1st–99th percentile) for all responders (fold–index ≥4) were 43.9–592.0 BAU/mL. A preliminary cut-off of 50 BAU/mL was set based on the percentiles of all responders and the convenience of manufacturing the WHO IS. The final cut-off value will be determined in future clinical trials.

Both our and Zhang’s data showed that approximately 10% of the studied population did not respond well to the inactivated SARS-CoV-2 vaccine following two doses, indicating the importance of monitoring poor or non-responders in this population [27]. Similar to Zhang’s report, sex affecting anti-spike IgG levels among the 18–59-year group after complete vaccination was also observed in our study due to females with higher estradiol hormone [27]. However, Zhang et al. did not report data on older adults. Our data showed no significant difference in the quantitative levels of anti-spiking IgG between the male and female groups aged ≥60 years (*p* = 0.4517), suggesting that female estradiol levels decline over the age of 60 years.

The WHO IS demonstrated the ability to compare antibody titers between different types of vaccines. Zitt et al. reported that the median titers of poor or non-seroconversion and seroconversion were 635.5 and 1565.0 BAU/mL after two doses of mRNA vaccination in hemodialysis patients at 67.6 ± 14.8 years, respectively [36]; whereas we reported that the median titers of poor or non-seroconversion and seroconversion were 63.9 and 346.0 BAU/mL after giving two doses of inactivated SARS-CoV-2 vaccines at 67 ± 6 years, respectively, indicating that antibody titers induced by the mRNA vaccines are higher than that of the inactivated SARS-CoV-2 vaccines [37].

The most significant benefit of PVST is that it saves patients’ lives. There were 2.1% severe outcomes for COVID-19 breakthrough infection in immunocompromised patients after two vaccine doses [8]. However, we can identify these with anti-spike IgG below 50 BAU/mL to reduce mortality. Chukwu et al. reported clinical findings in a group of kidney transplant recipients (KTRs) who received two doses of vaccines (72% of BNT162b2, 28% of AZD1222). There were 22 breakthrough infections and three deaths after vaccination, including 77% (17/22) infections and 13.6% (3/22) deaths in the seronegative group and only 23% (5/22) infections and 0% (0/22) deaths in the seropositive group [18]. Chavarot et al. reported that administration of a third dose of the BNT162b2 vaccine did not improve immunogenicity in KTRs treated with belatacept without prior COVID-19. Seropositivity was only 37.1% (13/35) of KTRs after the third vaccine dose. Twelve KTRs developed symptomatic COVID-19 after vaccination, with severe outcomes (50% of mortality) [38], although it is unclear whether poor or non-response to the vaccine will cause a breakthrough infection. Furthermore, these studies did not use the WHO IS to obtain a cut-off for the responder.

For SARS-CoV-2 vaccine-poor or non-responders, one benefit from PVST to the patient is to get a booster shot as soon as possible [39,40]. For persistently poor or non-responders to SARS-CoV-2 vaccination, anti-SARS-CoV-2 immunoglobulin injections could save these lives in the seronegative group following vaccination [41,42,43].

Another good example of PVST is the hepatitis B vaccine. After the first hepatitis B vaccine was approved in the United States in 1981 and the recombinant hepatitis B vaccine was approved by the FDA in 1986, it took scientists many years to realize that the vaccine did not provide good protection for the elderly and certain immunocompromised populations and put them at risk of breakthrough infections after vaccination [44,45]. Szmuness et al. have reported that 7.4% of immunized individuals fail to elicit detectable specific antibodies after two doses of the hepatitis B vaccine, suggesting that there are non-responders in the population [15]. Roome et al. have found that 11.9% of individuals with the hepatitis B vaccine had no or inadequate levels of antibodies, suggesting that PVST should be done at intervals of 30 to 90 days after the last vaccine dose [16]. Non-responders to hepatitis B vaccines were observed in adults, infants, as well as in drug users [13,46,47]. Many subsequent studies have shown that older adults and immunocompromised populations are associated with reduced vaccine responses to hepatitis B vaccination [45,46,47]. The CDC has recommended PVST using the WHO IS for immunocompromised individuals following the hepatitis B vaccine based on evidence of non-responders in the population [45]. For persistent non-responders (anti-HBs antibody < 10 mIU/mL, the WHO IS 07/164) to hepatitis B vaccination, anti-hepatitis B immunoglobulin injections are recommended if exposed to the hepatitis B virus [45].

The benefits of PVST outweigh the potential risks. Zitt et al. reported there was a median titer of 1440 BAU/mL in documented hepatitis B virus vaccine responders (anti-HBs antibody ≥10 mIU/mL) and a median titer of 308.5 BAU/mL in poor or non-responders (anti-HBs antibody <10 mIU/mL) after two doses of mRNA vaccination (*p* = 0.035), suggesting that PVST might predict the general immune competence [36]. All anti-SARS-CoV-2 spike IgG-positive patients recovered from the infection respond well to the vaccine, which indirectly proves this phenomenon [29,30]. If this theory turns out to be correct, then it might be possible that SARS-CoV-2 vaccine responders with normal immune cell counts have a strong ability to produce antibodies against variants through asymptomatic infections or cross-reactivity [24]. This may support the government-issued "immunity passports" to demonstrate an individual’s immune ability according to the WHO IS (≥ 50 BAU/mL) after recovering from COVID-19 or following the SARS-CoV-2 vaccination.

Furthermore, lower baseline immunity may be a major risk factor for poor or non-responders after the administration of the full SARS-CoV-2 vaccine in our study. Van Oekelen et al. have demonstrated that 32.3% (10/31) of multiple myeloma patients with severe lymphopenia (<500/mm^3^) remained negative for SARS-CoV-2 spike IgG after two doses of mRNA vaccines (OR 2.89, 95% CI 1.10–7.20, *p* = 0.018) [48]. Similarly, two studies reported that 63.7–77.3% of patients with a history of anti-CD20 therapy for B cell depletion remained negative for SARS-CoV-2 IgG after receiving mRNA vaccines, suggesting that B cells are required for humoral immunity following COVID-19 vaccines [49,50]. Our data showed that in the non-seroconversion group, there were 37.0–58.1% with low lymphocyte count (<1000/mm^3^), 33.3–45.2% with low CD4 cell counts (<500/mm^3^), and 74.1–96.8% with low B cell counts (<100/mm^3^), suggesting that lymphopenia, low CD4 cell counts, and low B cell counts were risk factors for poor or non-responders to vaccines. Presumed that specific cellular immunity in some immunocompromised individuals might be equivalent to that in healthy individuals, identification of poor or non-responders to the SARS-CoV-2 vaccine would still benefit these individuals. Hence, further clinical trials must be performed to finalize effective booster shots for immunocompromised patients after administering the complete dose to the general population [51,52,53,54,55].

Existing data show that laboratory testing has certain guiding significance for the prevention and treatment of COVID-19: (1) a normal immune cell and a good response to the SARS-CoV-2 vaccine might indicate healthy individuals; (2) a decrease in immune cells might predict disease severity and severe outcomes [6,56]; (3) anti-SARS-CoV-2 spike IgG levels <50 BAU/mL following the vaccine might indicate poor or non-response to the SARS-CoV-2 vaccine. According to this study, certain naïve immune cells, such as CD4, CD8, and B cells, exhibited significant waning in the elderly, suggesting that non-seroconversion rates were higher in individuals with lower baseline immunity. Our data showed that 7.5–11.7% of poor or non-responders existed in the population, supporting an FDA EUA quantitative assay with the WHO IS (20/136) cutoff might help to address this issue [25,26,57].

Approximately 10% of the studied population had poor or no response to both hepatitis B vaccines and SARS-CoV-2 vaccines, which is the content of urgent research to identify poor or non-responders in the population. Currently, among the people who have received two doses of the SARS-CoV-2 vaccine worldwide, many still have not received the third dose in children, older adults, and immunocompromised persons. We need to identify poor or non-responders using PVST as soon as possible because they are susceptible, and we need to prioritize the third dose. If PVST is not performed, these poor or non-responders might become vulnerable and easily develop severe COVID-19. However, there are several potential strategies that can be employed to reduce the COVID-19 mortality rate below 0.13% of that caused by the influenza virus. These include the following measures: (1) increase the vaccination rate of the general population [2]; (2) develop vaccines and/or anti-SARS-CoV-2 immunoglobulins against emerging and potential variants [58,59,60,61,62]; (3) administer booster vaccines for poor or non-responders [63,64]; (4) accelerate clinical trials of intranasal SARS-CoV-2 vaccines to prevent transmission [65]; (5) assess the specific B cell or humoral immune response of children, older adults, and immunocompromised persons within 14–90 days after vaccine booster shot to address concerns about vaccination hesitancy and refusal of vulnerable populations [45,66,67,68,69,70,71]; and (6) incorporate additional protective measures for individuals with persistent (a fourth or fifth dose) negative specific B cell or humoral immune response after booster vaccination, such as injection of anti-SARS-CoV-2 cross-reacting spike-specific immunoglobulins or variant spike-specific antibodies, antiviral drug treatment, and usage of N95 masks in endemic areas [41,42,43,72,73,74,75,76,77,78].

This study has some limitations. This single-center study analyzed demographics, including age and sex; however, race, religion, income, education, employment, and marital status were not included in the study. Although the precise matching of time after vaccination of each dose among the young and older adult population, sex or comorbid condition discrepancies still exist, which might contribute to a biased understanding of the result, which should be carefully avoided in future study designs. The WHO IS antibody levels can explain the clinical phenomenon of breakthrough infection, but why these phenomena occur requires the support of cellular immune data. The limited sample size hampered further exploration of potential concomitant changes in RBD/spike-specific cellular immunity, mucosal immunity, and humoral responses to the second dose. A larger prospective cohort to study cellular responses to booster doses of inactivated vaccines and compare the relative immune responses elicited by different platforms of SARS-CoV-2 vaccines is essential.

## 5. Conclusions

In this prospective clinical study, naïve immune cells, such as CD4, CD8, and B cells, and anti-spike IgG levels were significantly reduced in the elderly. There were 7.5% poor or non-responders to SARS-CoV-2 vaccines in the 18–59-year group and 11.7% poor or non-responders in the ≥60-year group. The effective seroconversion rate was significantly related to the level of certain naïve immune cells before vaccination, such as total lymphocytes, CD4 and B cells, as well as age. There were 37.0–58.1% with lymphopenia (<1000/mm^3^), 33.3–45.2% had low CD4 cell counts (<500/mm^3^), and 74.1–96.8% had low B cell counts (<100/mm^3^) in the non-seroconversion group. Sex affected anti-spike IgG levels in the 18–59-year group after standard two-dose SARS-CoV-2 vaccines. There were no significant differences in the quantitative level of anti-spike IgG at different time points (14–30, 31–60, and 61–90 days) after administering two doses of the vaccine. An individual with an anti-SARS-CoV-2 spike IgG titer below 50 BAU/mL might be considered a poor or non-responder between 14 and 90 days after the last vaccine dose.

## Figures and Tables

**Table 1:** Immunological characteristics of individuals before and after vaccination.

Characteristics	18–59-Year Group	≥60-Year Group	*p*-Value
Total number of cases	361	266	
Sex (%)			
Male	183 (50.7)	136 (51.1)	
Female	178 (49.3)	130 (48.9)	0.1201
Age, yrs mean (SD)	45 (9)	67 (6)	<0.0001
Naïve immune cells, median (IQR)			
Lymphocytes (/mm^3^)	1476 (1168–1875)	1281 (1023–1520)	<0.0001
CD4 cells (/mm^3^)	851 (677–1151)	747 (562–955)	<0.0001
CD8 cells (/mm^3^)	490 (357–632)	418 (288–544)	<0.0001
B cells (/mm^3^)	256 (179–367)	204 (138–303)	<0.0001
Natural killer cells (/mm^3^)	193 (141–287)	234 (162–355)	<0.0001
Anti-spike IgG			
Titer, BAU/mL, median (IQR)	416.8 (355.7–479.2)	307.2 (118.2–417.3)	<0.0001

SD, standard deviation; IQR, interquartile range; IgG, immunoglobulin G; CD, cluster of differentiation.

**Table 2:** Characteristics of immunity before and after the complete vaccination.

Characteristics	18–59-Year Group			≥60-Year Group		
Groups	Fold-Index < 4	Fold-Index ≥ 4	*p*-Value	Fold-Index < 4	Fold-Index ≥ 4	*p*-Value
Total number of cases	361			266		
Anti-spike IgG, BAU/mL (the 2.5th–97.5th percentile)	88.9–576.2			27.7–491.0		
Fold–index, % (no.) *	7.5 (27/361)	92.5 (334/361)		11.7 (31/266)	88.3 (235/266)	
Anti-spike IgG, BAU/mL						
Median (IQR)	115.8 (88.6–167.8)	420.8 (369.9–480.6)	<0.0001	63.9 (35.1–106.9)	346.0 (160.4–424.7)	<0.0001
The 2.5th–97.5th percentile	11.3–266.3	200.7–576.5		5.4–317.8	46.6–491.1	
Naïve immune cells (/mm^3^)						
Lymphocytes, mean (95% CI)	1130 (1007–1252)	1578 (1524–1633)	<0.0001	1015 (888–1143)	1344 (1291–1397)	<0.0001
CD4 cells, mean (95% CI)	631 (555–708)	942 (905–979)	<0.0001	563 (494–631)	818 (777–858)	<0.0001
CD8 cells, mean (95% CI)	414 (349–479)	532 (508–557)	0.0081	394 (310–478)	444 (420–468)	0.1744
B cells, mean (95% CI)	119 (72–166)	306 (289–323)	<0.0001	74 (60–88)	248 (231–266)	<0.0001
NK cells, mean (95% CI)	192 (151–233)	235 (220–251)	0.1241	281 (225–337)	286 (261–311)	0.8902

* Post-vaccination testing is performed 14–90 days after the second vaccine dose. The reference range is defined as the 2.5th–97.5th percentile in this study. NK, natural killer; IQR, interquartile range; IgG, immunoglobulin G; CD, cluster of differentiation; CI, confidence interval; SARS-CoV-2, severe acute respiratory syndrome coronavirus 2.

**Table 3:** Baseline characteristics of immune cells in the non-seroconversion group.

Characteristics *	18–59-Year Group	≥60-Year Group
Lymphocytes (<1000/mm^3^)	37.0%	58.1%
CD4 cells (<500/mm^3^)	33.3%	45.2%
CD8 cells (<150/mm^3^)	0%	3.2%
B cells (<100/mm^3^)	74.1%	96.8%
NK cells (<70/mm^3^)	3.7%	0%

* In the non-seroconversion group (fold-index <4). Lymphopenia is defined as a lymphocyte count <1000/mm^3^. NK, natural killer.

## Data Availability

The data that support the findings of this study are available from the corresponding author upon reasonable request.

## Supplementary Materials

The following supporting information can be downloaded at: https://www.mdpi.com/article/10.3390/vaccines11020461/s1, Figure S1: A schematic overview illustrating participant enrollment.
