# Supplementary figures and images for: Immunological Findings in a Group of Individuals Who Were Poor or Non-Responders to Standard Two-Dose SARS-CoV-2 Vaccines
Source: Vaccines (Basel). 2023 Feb 16;11(2):461. doi: 10.3390/vaccines11020461 (PMC9963224; doi:10.3390/vaccines11020461)

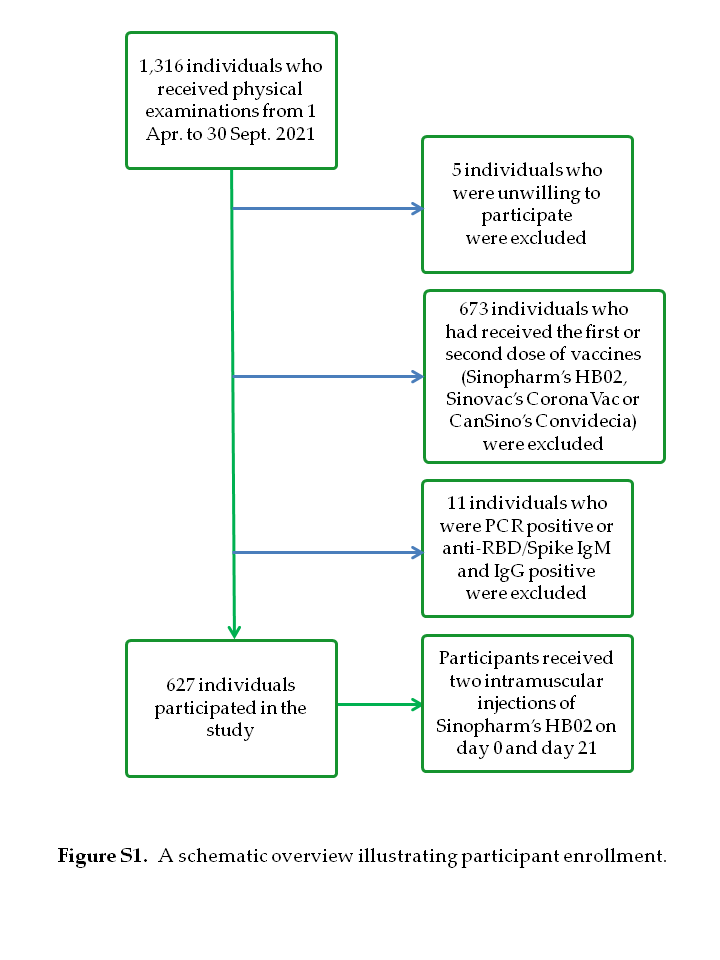

Supplement: Supplementary file 1 [file vaccines-11-00461-s001.zip › vaccines-2121156-supplementary.gif]
